# Phenotypic and genotypic detection of metallo-beta-lactamases in Carbapenem resistant Acinetobacter baumannii

**DOI:** 10.22088/cjim.11.2.171

**Published:** 2020

**Authors:** Zahra Moulana, Arefeh Babazadeh, Zohreh Eslamdost, Mehran Shokri, Soheil Ebrahimpour

**Affiliations:** 1 Infectious Diseases and Tropical Medicine Research Center, Health Research Institute, Babol University of Medical Sciences, Babol, Iran; 2 Department of Microbiology, Yahyanejad Hospital, Babol University of Medical Sciences, Babol, Iran

**Keywords:** *Acinetobacter baumannii*, Carbapenem resistance, Metallo-beta-lactamases, Modified hodge test

## Abstract

**Background:**

Carbapenem resistance in *Acinetobacter baumannii *has become a major concern for treating physicians. The aim of this study was to investigate the prevalence of metallo β-lactamase (MBL) genes (*bla *_VIM_* , and bla*_IMP_) among isolated multidrug-resistant *A. baumannii* .

**Methods:**

Fifty non-repetitive carbapenem-resistant *A. baumannii* isolates were collected. Antibiotic susceptibility was performed by disk diffusion method. MICs were determined by E test method. The resistant strains were tested for the production of carbapenemases by the Modified Hodge Test (MHT) followed by EDTA-disk synergy test was performed for metallo-β-lactamases (MBL) phenotypic detection. Detection of *bla *_VIM_ , and *bla*_IMP_ was performed by PCR followed by sequencing.

**Results:**

All isolates had a multidrug resistant profile, and were all resistant to all antibiotics including the carbapenems but remained susceptible to colistin. Among these isolates, Carbapenemase production was confirmed by the Modified Hodge test for 42 (84%) isolates. Phenotypic method showed the production of MBL in 15 (30%) isolates. PCR techniques revealed that out of 50 isolates, 13 (26%) were positive for *bla*_VIM_ and all were negative for *bla*_IMP_*.*

**Conclusion:**

Our study concludes that the high prevalence of carbapenem resistant *Acinetobacter *species with MBL production is one of the main concerns in our country and this situation needs strict infection control measures.

*TAcinetobacter* *baumannii* is a gram-negative coccobacillus initially considered to be an opportunistic pathogen, which plays a vital role as a major cause of healthcare-associated infections (1, 2). In recent years, *Acinetobacter *has become resistant to most effective antimicrobial agents and causing a high incidence rate of morbidity and mortality especially in the intensive care unit in many countries (3, 4). The emergence of multidrug-resistant (MDR: resistant to at least one agent in three or more antimicrobial categories or to one key treatment antibiotic), extensively drug-resistant(XDR: resistant to at least one agent in all but two or fewer antimicrobial categories) or pandrug-resistant (PDR: resistant to all agents in all antimicrobial categories) isolates causes serious problems in treating *Acinetobacter* infections (4, 5). Carbapenems are considered the most effective drugs for the treatment of infections caused by multidrug- resistant gram negative bacteria, when these bacteria are resistant to other β-lactam antibiotics.(6). Unfortunately, many studies have reported high carbapenem resistance rates among these pathogens from different countries and this situation expresses serious therapeutic challenges (7, 8)*.*

The ability to produce Carbapenemase enzymes such as oxacillinases (Ambler class D *OXA*-type) and metallo-β-lactamases (MBLs) (Ambler class B) are the most frequent resistance mechanisms in A. baumannii (9, 10). Since resistance to carbapenems is difficult to detect by routine disc diffusion method (11), various inhibitin-based tests such as the double-disk synergy test, combined disk test and the modified Hodge test (MHT) have been recommended by CLSI as general phenotypic methods for detection of carbapenemases (12, 13). Also, polymerase chain reaction (PCR) is used as a reliable method for the identification of the most prevalent genes in carbapenemase positive clinical isolates (14). This study aimed to detect the frequency of carbapenemase and MBL producing MDR A. baumannii by phenotypic methods and the detection of the bla_VIM _and bla_IMP _genes by (PCR) in patients of hospitals affiliated with Babol University of Medical Sciences.

## Methods

Fifty non-duplicate carbapenem resistant *Acinetobacter *isolates were collected from several units of Babol University of Medical Sciences affiliated hospitals, Babol, Iran, from January 2015 to June 2016. The most common sources of strains were isolated from respiratory samples (endotracheal aspirates, sputum), ulcers, urinary specimens and blood. Samples were cultured on bacteriological blood agar and eosin methylene blue (EMB) media (Merck, Germany) and incubated in aerobic condition at 37ºC for 24 hours. The colonies were identified up to the species by specific biochemical tests using API 20E strips (bioMérieux, Marcy l’Etoile, France) according to the manufacturer's instructions. Strains were stored at -80°C for molecular analysis.

**Antimicrobial Susceptibility Testing and determination of MIC: **The sensitivity of different classes of antimicrobial agents was determined using disk diffusion method according to CLSI guidelines (2015). The following antibiotics were used; cefazidime (CAZ :30 µg), colsitin (Col:110 µg), cefepime (FEP: 30 µg), imipenem (Imp :30 µg), meropenem (MER: 30 µg), ciprofloxacin (CIP: 5 µg), ceftriaxone (CRO:30 µg), piperacillin-tazobactam (PTZ: 100/10 µg), trimethoprim-sulfamethoxazole (SXT :1.25/23.75 µg) ,Gentamycin (GM: 10 µg), ampicillin/ sulbactam (SAM: 10/10 µg) and ertapenem (ETP: 10 μg) (MAST, UK).* Escherichia coli *ATCC25922 and *Pseudomonas aeruginosa *ATCC 27853 strains were used as negative control strains. MBL-producing *P. aeruginosa* and carbapenemase-producing *A. baumannii* were used as positive control strains. In addition, Minimum Inhibitory Concentrations (MICs) were determined by E-test MIC strips (BD, France, and Liofilchem R) .The concentration ranges for the *E*-test was 0.002–32 µg/ ml for the carbapenems (BD,France, and Liofilchem R ).

**Screening for the Carbapenemase Production: **All isolates with reduced susceptibility to meropenem and imipenem (diameter of zones of inhibition ≤13mm) by disc diffusion method were tested for the presence of carbapenemase by Modified Hodge test (MHT) using a meropenem disc (10 μg) as per CLSI guidelines. The quality strain used is *E. coli* ATCC 25922. Also, the double disk synergy test (DDST) for the ability of the detection of MBL possessing isolates (15), and compared to PCR for the detection of MBL gene *bla*_IMP _and *bla*_VIM._

**DNA extraction and amplification of carbapenemas genes: **DNA was extracted using a kit supplied by Roche, (Roche Diagnostics,Germany). PCR ampliﬁcation for the detection of *bla*_VIM _and *bla*_IMP_ genes were carried out on a thermal cycler (Eppendorf, Hamburg, Germany). The sequences of used primers for the amplification of the *bla*_VIM _gene were F:5'-AGTGGTGAGTATCCGACAG-3' and R:5'-TGAAAGTGCGTGGAGAC-3' which produce a 261bp PCR product. The sequences of primers used for amplification of *bla*_IMP_ gene were F:5'-TCGTTTGAAGAAGTTAACGG-3' and R:5'-ATGTAAGTTTCAAGAGTGATGC-3' which are known to give a PCR product of about 568bp. The PCR reaction was performed in 50 μL volumes that contained 10 µL extractions of DNA (equal to 1 μg), 5 pmol/L from each primer, 1.5 mmol/L MgCl2, 0.2 mmol/L dNTPs (Fermentas, GmbH, Germany) and 1.5 unit of Taq DNA polymerase enzyme. PCRs were run using the following steps: primary denaturation at 94 ºC for 5 minutes followed by 30 cycles of denaturation at 94 ºC for 25 seconds, annealing at 52 ºC for 40 seconds and extending at 72 ºC for 50 seconds. Moreover one cycle for the final extension at 72 ºC for six minutes was performed. Then, 10μL of the PCR products was conducted in 1.5% agarose gel (Cinagene Co, Iran) stained by 0.5µg of ethidium bromide/ml (Sigma, Germany) and the results were evaluated in the presence of 100 bp. DNA size marker (Fermentas Co, Ukraine), visualized under UV trans illuminator. Finally, amplification products were sequenced by Macrogen Inc, Seoul, Korea. The collected data were statistically analyzed using SPSS program (software Version 17.0).

## Results

**Figure 1:**
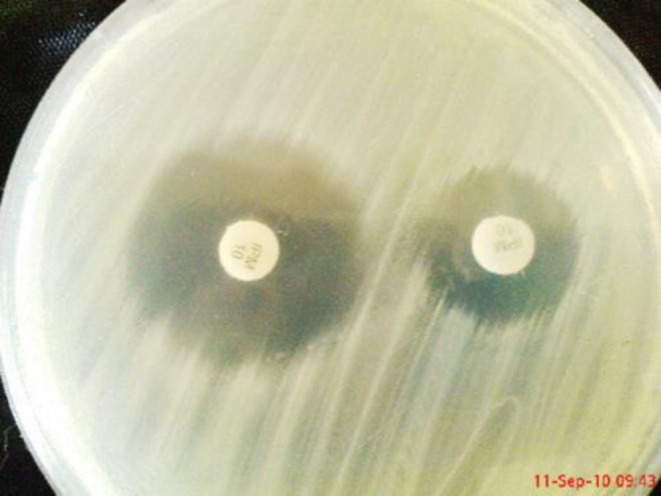
Phenotypic detection of metallo-beta-lactamases by combined disk test and double disk synergy test

From 50 carbapenem-resistant *A. baumannii *isolates, 30 (60%) were collected from female patients. The mean age of patients was 65.25 years and 44 (88%) were aged 50 years and older. The highest number of these isolates were found in patients hospitalized in intensive care units (ICUs) 34 (68%), followed by infectious disease units 12(24%) and surgical clinics 4 (8%). The most common sources of these strains were isolated from respiratory samples (endotracheal aspirates, sputum) 30 (60%), ulcers 12 (24%), urinary specimens 6 (12%) and blood 2 (4%), respectively. It should be noted that 46 (92%) of patients recovered and were eventually discharged with good general condition and 4 (8%) patients passed away.All isolates had a multidrug resistant profile, and were all resistant to all antibiotics including the carbapenems. The carbapenem resistance profile of all isolates was confirmed by determining the MIC value of meropenem by the E-test method. The only class of antibiotics to which the isolates were susceptible was colistin sulfate (94%) and trimethoprim-sulfamethoxazole (14%). The results of the antimicrobial susceptibility testing showed that colistin was the most effective in vitro antimicrobials against the majority of *A. baumannii* isolates. Only three isolates were resistant to colistin. Moreover for meropenem and imipenem, the result of the E-test was consistent with the disk diffusion method and 100% of isolates were resistant by the two methods. Among these isolates, carbapenemase production was confirmed by the modified Hodge test for 42 (84%) isolates (figure 1). 

**Figure 2:**
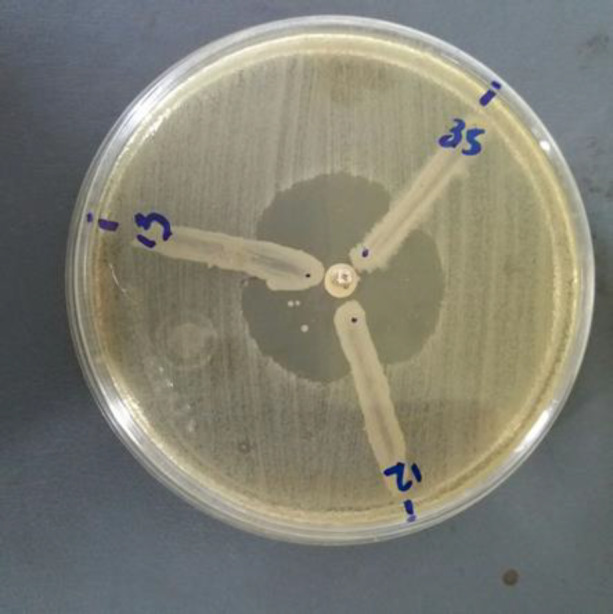
The MHT on a 100 mm MH plate strain positive result

**Figure 3:**
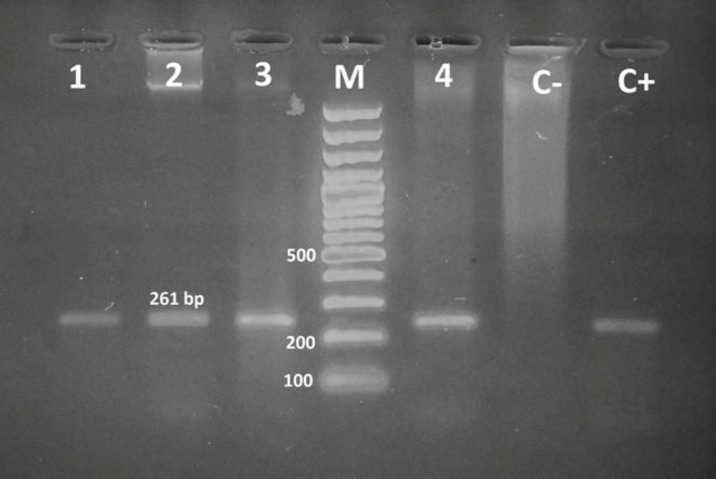
Agarose gel electrophoresis of PCR amplified productsof Acinetobacter baumannii blaVIM MBLs gene: Lane 1 -3, 4: positive isolate, Lane M: DNA size marker, Lane C+ : positive control, Lane C-: negative control

The phenotypic method showed the production of MBLs in 15 (30%) isolates (figure 2). Searching for MBLs genes in all isolates by PCR technique revealed that out of 50 isolates, 13 (26%) were positive for *bla*_VIM_ and all of them were negative for *bla*_IMP_ (figure 3).

## Discussion

In the present study, *A. baumannii *were isolated most commonly from respiratory tract specimens, followed by wounds and urine. Many studies also reported that respiratory secretion specimens are the major source of *A. baumannii *isolates, followed by wounds (6, 16). In the past decad,e many reports have indicated that the frequency of MDR *A. baumannii* has to increase from 50% in 2001–2007 to 74% in 2010–2015 among the hospitalized patients in Iran (17, 18). The prevalence rate of A. baumannii isolates from other neighboring countries, including Turkey , Pakistan, Emirates and Saudi Arabia are similar to the antimicrobial profile reported in Iran (19, 20). Carbapenems are generally used as the last choice in the treatment of MDR gram-negative bacterial infections (21). In accordance with previously performed studies, antibiotic susceptibility and MIC tests in the current work showed that 94% of *A.baumannii* isolates were found to be PDR, which indicate resistance to all classes of antibiotics except colistin. However, some studies have reported that *A. baumannii* isolates are becoming resistant to colistin (22, 23). In our study, we found high frequency resistance to imipenem and meropenem, which correlates with previously reported studies (24). In view of the increasing resistance to carbapenems, this study also highlights phenotypic tests such as the modified Hodge test for a first-line detection of carbapenemase-producing isolates (25, 26). Results from the MHT in the current study showed that 84% of *A.baumannii* isolates were carbapenemase producers. These results are supported by several studies which have found that the MHT is a useful screening test for Carbapenemase production (27, 28). According to the findings of several studies, the use of DDST is one of the more reliable methods for the detection of Ambler class B MBL production with a high rate of positivity (29). 

Furthermore, our study has shown the lowest positivity rate (30%) by DDST method and our result are similar to a study conducted by Shivaprasad et al (30). The results of the present work show that the phenotypic and genotypic results of the detection of MBL producing isolates were similar, and that the PCR assay of all carbapenem resistant isolates revealed that 26% (13/50) of isolates carried the *bla*_VIM_ gene. In the current study, the *bla*_IMP_ gene was not detected among the *A. baumannii* isolates. In a previous study conducted by Fallah et al., results showed that the prevalence of the *bla*_VIM_ and *bla*_IMP _gene in *A. baumannii *were 17% and 4%, respectively(31). In a study conducted by Erfani et a,l. results showed that the prevalence of the *bla*_VIM_ gene in *A. baumannii* is 60.4% while no *bla*_IMP_ positive cases were reported (32). In another study from Iran in 2018, the prevalence of *bla*_VIM_ and *bla*_IMP_ genes was found to be 62.3 and 30.4 %, respectively (33).

In conclusion this study confirmed the high prevalence of multidrug-resistant *A. baumannii* isolates in the ICU, which is emerging as a predominant pathogen in hospitals. In case of multidrug-resistant *Acinetobacter* infections, colistin is the best choice of antibiotic available for the effective treatment of serious infections. Initial screening of the putative carbapenemase producers will help to manage infection-control policy and early directed therapy. Easy detection methods are required for routine clinical labs. Our study showed that MHT and E-test were equally efficient to detect MBL production, followed by combined disc test. The use of molecular methods is essential, especially in limiting the spread of infection.
